# A modified oxic-settling-anaerobic activated sludge process using gravity thickening for excess sludge reduction

**DOI:** 10.1038/srep13972

**Published:** 2015-09-09

**Authors:** Jun Wang, Shi-Yu Li, Feng Jiang, Ke Wu, Guang-Li Liu, Hui Lu, Guang-Hao Chen

**Affiliations:** 1 Guangdong Provincial Key Laboratory of Environmental Pollution Control and Remediation Technology, School of Environmental Science and Engineering, Sun Yat-sen University, Guangzhou 510275, China; 2 School of Chemistry and Environment, South China Normal University, Guangzhou, China; 3 Department of Civil & Environmental Engineering, The Hong Kong University of Science and Technology, Clear Water Bay, Kowloon, Hong Kong, China

## Abstract

Oxic-settling-anaerobic process (OSA) was known as a cost-effective way to reduce the excess sludge production with simple upgrade of conventional activated sludge process (CAS). A low oxidation-reduction potential (ORP) level was the key factor to sludge decay and lysis in the sludge holding tank of the OSA process. However, the ORP control with nitrogen purge or chemical dosing in the OSA process would induce extra expense and complicate the operation. Hence, in this study, a sludge holding tank using gravity thickening was applied to OSA process to reduce the excess sludge production without any ORP control. Results showed that the modified OSA process not only reduced the excess sludge production effectively but also improved the sludge settleability without affected the treatment capacity. The reduction of the excess sludge production in the modified OSA process resulted from interactions among lots of factors. The key element of the process was the gravity thickening sludge holding tank.

Activated sludge processes play the major role in biological wastewater treatment through microbe-mediated conversion of biodegradable organic and nutrient pollutants into gaseous and bio-solids while unbiodegradable matter ends up in excess sludge. About 3,000,000 tons of dewatered sludge has been produced daily in China^1^,^2^. The treatment of excess sludge accounts for 40–60% of the total cost of an activated sludge treatment plant and the place for ultimate disposal such as landfill or incineration is limited^3^. To solve the issue, an oxic-settling-anaerobic process (OSA), which is a modification of a conventional activated sludge process (CAS) with anoxic/anaerobic treatment of returned sludge by inserting a sludge holding tank in the sludge return line, has been developed and proved to be an effective *in situ* approach to control the excess sludge production^3^,^4^,^5^,^6^,^7^,^8^,^9^,^10^,^11^. With almost 30–50% reduction of excess sludge, the OSA process offers a cost-efficient solution to control the sludge production because neither chemical/physical pretreatment of excess sludge nor metabolic inhibitor is needed.

Nevertheless, applications of OSA process are still limited. This is mainly attributed to a low oxidation-reduction potential (ORP) requirement in the sludge holding tank. Our previous studies suggested that sludge decay with the aid of a low ORP level in the sludge holding tank could be the major cause of the reduction in excess sludge in the OSA process^6^. Under an ORP level of −250 mV, sludge decay, disintegration and solubilization could be accelerated effectively^7^. This requirement, however, will induce extra expense and complicate the operation. Because ORP control in an OSA process is usually associated with injection of pure nitrogen gas^6^,^7^,^9^ or dosing with reductant^3^.

To solve the problem, the first-order kinetics applied for sludge decay process^12^,^13^,^14^,^15^,^16^,^17^,^18^,^19^,^20^,^21^ suggested a new way to improve sludge decay without ORP control in the sludge holding tank, which is increasing the sludge concentration to a higher level to achieve a higher sludge reduction.


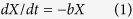


Where

X = concentration of the biomass(mg/l)

b = decay coefficient(d^−1^)

After integration the decayed biomass can be expressed as:


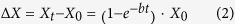


Where

ΔX = the decayed biomass(mg/l)

X_t_ = biomass concentration over time(mg/l)

X_0_ = initial biomass concentration(mg/l)

The decayed biomass is proportional to the initial biomass concentration. More sludge will decay and more substrate will be produced due to higher initial biomass concentration. It implies that a high sludge concentration in the sludge holding tank would help sludge decay. Meanwhile, the products of sludge decay would provide the substrate for the low-yield anaerobic/anoxic reactions.

Hence, in this paper, a modified OSA system using gravity thickening sludge holding tank was developed to investigate the sludge reduction performance as well as organic, nitrogen and phosphorus removal efficiencies.

## Results

### Excess sludge production

**Figure 1:**
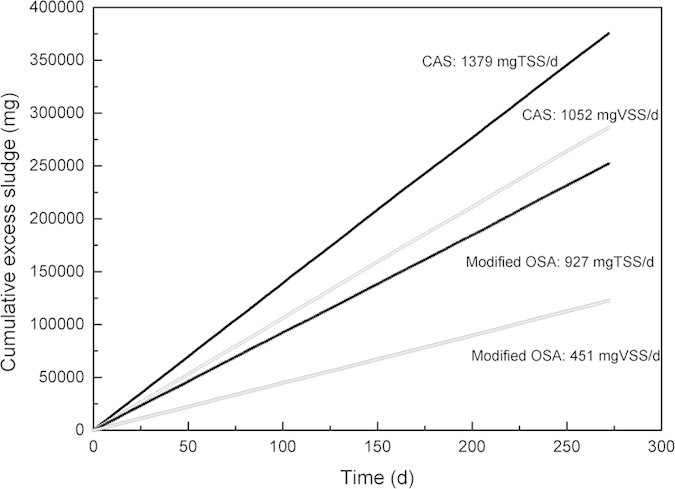
Cumulative sludge production in the CAS and the modified OSA processes.

Figure 1 shows the cumulative excess sludge production during 272d in the CAS and the modified OSA processes after 2-month cultivation period. Since 0.7 l and 0.5 l excess sludge were withdrawn from the aeration tank of the CAS and the modified OSA processes to maintain the average total suspended solids (TSS) concentration at 1700 ∼ 1800 mg/l, the excess sludge production of the modified OSA process was 33% less than the CAS process calculated as TSS mass (927 and 1379 mgTSS/d respectively) or 57% calculated as volatile suspended solids (VSS) mass (451 and 1052 mgVSS/d respectively). The difference between the TSS and VSS production was attributed to the phosphorus accumulating organisms (PAOs) accumulation in the modified OSA system, although the PAOs enrichment was restricted. Due to the absorption of polyphosphate (PP), PAOs tended to have higher inorganic dissolved solids (IDS) content than ordinary heterotrophic organisms (OHOs)^22^.

Table 1 summarizes the comparison of the excess sludge reduction between different OSA processes. Although the reduction effect of the modified OSA process was lower than the normal OSA processes, the observed yield coefficient (Y_obs_) was almost the same. This was mainly because the normal OSA processes had a higher organic loading which would make the reduction effect more obvious. The sludge production of the CAS process will increase with the organic loading, while the organic loading had much less influence to the OSA process^5^. The Y_obs_ of the modified OSA process was 0.24 gTSS/gCOD, which was the same as the normal OSA processes of Chudoba *et al.*^5^ and Wang *et al.*^9^, but slightly higher than Saby *et al.*^7^. This was because that a hollow fiber membrane separation module was employed in Saby *et al.*^7^ which would extend the sludge retention time in the aeration tank and then reduce more sludge production^9^.

Therefore, without any external ORP control, the modified OSA process reduced the excess sludge production effectively.

### Treatment efficiency and SVI

**Figure 2:**
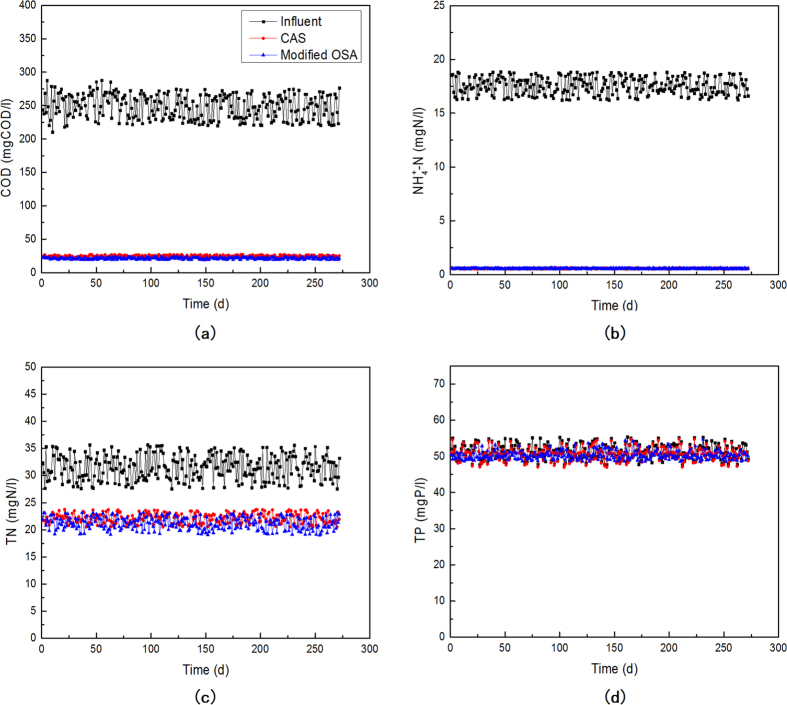
Removal performance of (a) COD, (b) NH_4_^+^-N, (c) TN and (d) TP in the CAS and the modified OSA process.

After the 2 months cultivation period, the performance of the CAS and the modified OSA processes became stable. Figure 2 shows the removal performance of chemical oxygen demand (COD), NH_4_^+^-N, total nitrogen (TN) and total phosphorus (TP) in both processes.

It was found that the average treatment efficiency of COD in the CAS and the modified OSA process was 90.4% and 91.6% respectively. And almost all NH_4_^+^-N could be removed in the CAS and the modified OSA process, the average treatment efficiency of NH_4_^+^-N was 96.7% in both processes. On the other hand, the insertion of the sludge holding tank offered a favorable condition for denitrification (DN) and PAOs enrichment. However, the substrate in the sludge holding tank was limited and the nitrate flowed into the tank was submitted to the recirculation flowrate, the effect of DN and PAOs enrichment were restricted. The average treatment efficiency of TN in the CAS and the modified OSA process was 33.3% and 36.3% respectively. And the average treatment efficiency of TP was 1.4% and 2.3% respectively.

However, the modified OSA process was found stimulated the growth of PAOs. The average phosphorus content of the modified OSA process sludge was 0.07 mgP/mgVSS, which was 3 times higher than that in CAS processes as 0.02 mgP/mgVSS^23^. In comparison, a typical phosphorus content in the sludge mass in an enhanced biological phosphorus removal (EBPR) process is about 0.06–0.15 mgP/mgVSS^23^. Therefore, the insertion of the sludge holding tank promoted the growth of PAOs. However, without an effective anaerobic P release process, the modified OSA process can not achieve high P removal rate. But these results implies that, the insertion of the sludge holding tank in an A^2^/O process may help not only in sludge reduction but also P removal.

**Figure 3:**
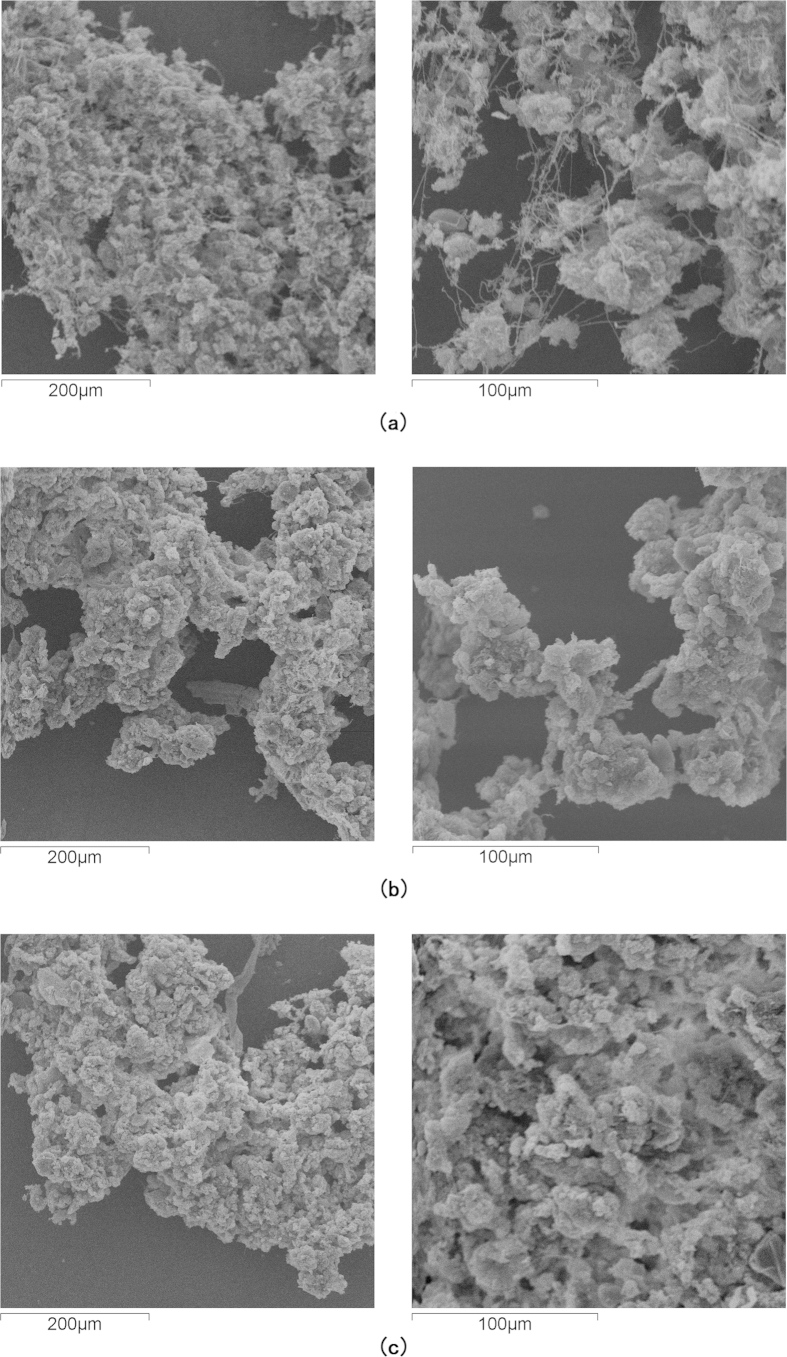
Scanning electronic microscopic photos of sludge from (a) the aeration tank of the CAS process, (b) the aeration tank and (c) the sludge holding tank of the modified OSA process.

**Figure 4:**
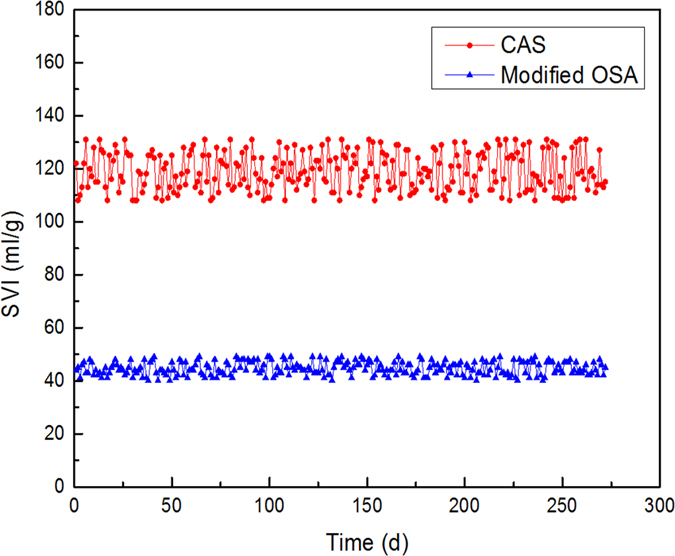
Variations of the SVI in the CAS and the modified OSA processes.

Apart from the maintaining of the removal performance in the modified OSA process, settleability was also found better than the CAS process and the normal OSA processes. Figure 3 shows the scanning electronic microscopic photos of the sludge from the CAS and the modified OSA processes. It was found that the structure of the sludge from the CAS process was loose and flat, the majority of the bacteria in the sludge were filamentous microorganisms, while the sludge from the modified OSA process had a strong sense of space and no filamentous microorganisms were found. Excessive growth of filamentous microorganisms will lead to bulking sludge, which is a common problem in activated sludge process^24^,^25^. Bulking sludge flocs are open and porous, the settling is hindered and associated with a high sludge volume index (SVI)^23^. Figure 4 shows the variations of the SVI in both processes. The average SVI in the CAS and the modified OSA process was 119 ml/g and 45 ml/g respectively. The SVI in a normal OSA process was about 50 ∼ 200 ml/g, depending on the ORP level^5^,^7^. Hence, the sludge holding tank in the modified OSA process played a role as an anoxic/anaerobic selector^24^ which would inhibit the growth of the filamentous microorganisms. Furthermore, PAOs could improve the settleability because they usually formed dense clusters^26^.

With the insertion of the sludge holding tank, the modified OSA process will not affect the treatment capacity but improve the sludge settleability and reduce the excess sludge production in the mean time.

### ORP level and sludge concentration distribution in the sludge holding tank

**Figure 5:**
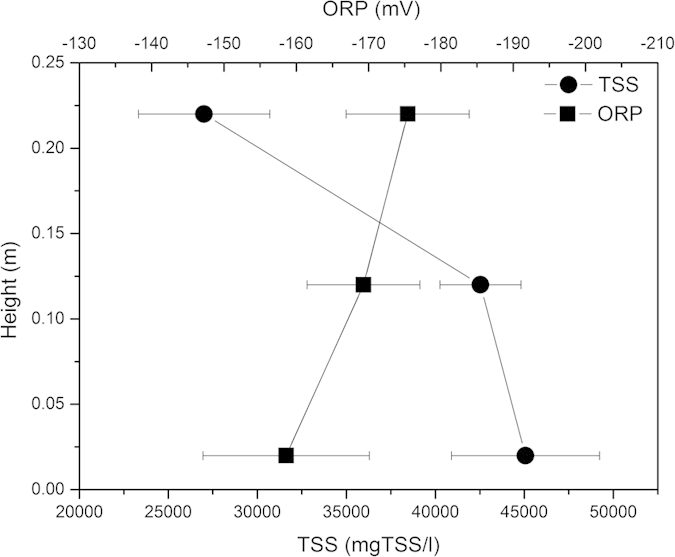
ORP level and sludge concentration distribution in the sludge holding tank.

Figure 5 shows the ORP level and the sludge concentration vertical profile in the sludge holding tank. The ORP level and the sludge concentration decreased gradually along vertical direction. The average ORP level dropped to −170 mV automatically without any ORP control. This could be explained by the fact that the ORP level was in direct proportion to oxidant concentration and inversely proportional to reductant concentration. As demonstrated above, the oxidant (e.g. O_2_ and NO_3_^−^) decreased and substrate released along vertical direction in the sludge holding tank. Therefore, the ORP level decreased gradually. However, due to the limited retention time in the sludge holding tank, the ORP level cannot drop to −250 mV.

On the other hand, due to the gravity thickening, the average sludge concentration in the sludge holding tank reached up to 43069 mgTSS/l, accounted for almost 92% of the total sludge mass in the modified OSA system. This value was much higher than a normal OSA process, whose average sludge concentration in the holding tank was about 8600 mgTSS/l^6^ or 4600 mgTSS/l^5^. Large amount of sludge accumulation inside the sludge holding tank made the SRT of the modified OSA system much longer, and rendered sludge under long-term endogenous processes, which would further promote the sludge reduction effect.

Thus, high sludge concentration in the sludge holding tank of the modified OSA process would help to reduce the ORP level and the excess sludge production effectively.

### Reactions in the sludge holding tank

**Figure 6:**
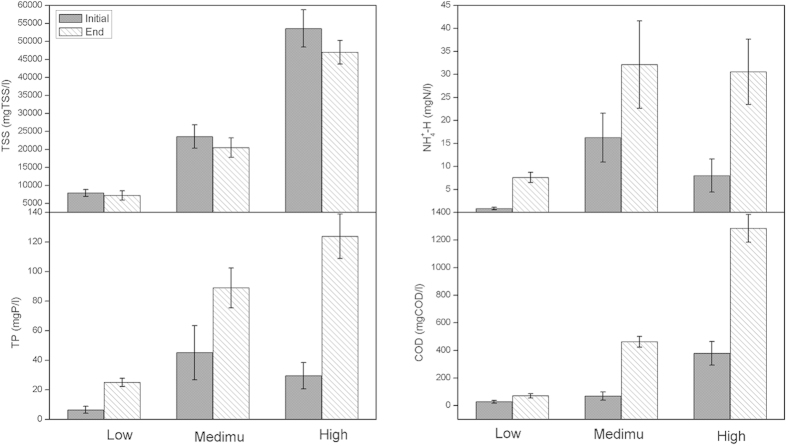
Impact of the sludge concentration to the decay behavior.

As mentioned above, sludge decay in the sludge holding tank could be the major cause of the reduction in excess sludge in the OSA process. Without any ORP control, the sludge holding tank which enriched a large amount of sludge using gravity thickening played a key role in sludge decay. To further investigate the mechanisms, batch tests were conducted to evaluate the effect of sludge concentration on sludge reduction and COD, TN and TP removal rates. Figure 6 shows the impact of the sludge concentration to sludge decay behavior. After 22 h anaerobic exposure of the modified OSA sludge, TSS decreased with COD, TP and NH_4_^+^-N released. The higher the initial TSS concentration, the more sludge would decay and the more substrate would release accordingly. These results agreed with the first-order kinetic equation applied for the decay process. The decay amount was proportional to the initial concentration.

Apart from sludge decay, substrate released and NO_3_^−^-N depletion were observed in the sludge holding tank of the modified OSA system, which indicated that hydrolysis and DN took place. The average COD, TP, and NH_4_^+^-N concentrations in the effluent of the sludge holding tank were 46 mgCOD/l, 73 mgP/l and 18 mgN/l respectively and no NO_3_^−^-N was detected, while the average COD, TP, NH_4_^+^-N and NO_3_^−^-N concentrations in the influent of the sludge holding tank were 21 mgCOD/l, 50 mgP/l, 0.6 mgN/l and 20 mgN/l respectively. Therefore, the main anoxic/anaerobic reactions involved in the modified OSA process were hydrolysis and DN. According to the principle of microbial metabolism^27^, anoxic and anaerobic reactions usually produced less biomass and more gas compare to aerobic reactions because more electrons from the electron donor would pass to the electron acceptor rather than biomass. In this way, the modified OSA process would generate less biomass and maintain the treatment capacity.

Therefore, the products of sludge decay offered the substrate for the anoxic/anaerobic reactions occurred in the sludge holding tank and so excess sludge would reduce and treatment efficiencies would increase accordingly.

## Discussion

The essence of biological wastewater treatment is elemental cycle, which means the pollutants in liquid will be converted to gas or solid by means of microbial metabolism. Therefore, to improve the gas production in an activated sludge process would help to reduce the excess sludge production.

With the aid of the gravity thickening sludge holding tank, a large amount of sludge was enriched in the modified OSA process, which would enormously extend the SRT and make sludge under long-term endogenous processes to improve sludge decay. Endogenous processes, which result from numerous mechanisms like: maintenance energy requirements, decay of cells, endogenous respiration, grazing by higher animals, or lysis due to adverse environmental conditions (pH, toxic substance, temperature or viral attack), were usually associated with the disappearance of suspended organic matter^28^. Besides, oxides rapid depletion and released of reducing substances brought down the ORP level in the sludge holding tank and would also accelerate sludge decay. The products of sludge decay provided the substrate for those low-yield anaerobic/anoxic reactions since almost all soluble substrate had already been utilized in the aeration tank and there was no external substrate supply in the sludge holding tank. The sludge decay under high sludge concentration due to gravity thickening and the consequent anaerobic/anoxic reactions in the sludge holding tank produced less biomass and more gas and, eventually, reduced the excess sludge. On another hand, the modified OSA process is also a selector-like system, the sludge are subjected to feast and famine environment, which would inhibit the growth of the filamentous microorganisms and then improve the settleability^29^,^30^,^31^.

In summary, without any ORP control, the modified OSA process not only worked well in sludge reduction, but also increased the sludge settleability without affected the treatment capacity. The reduction of the excess sludge production in the modified OSA process resulted from interactions among lots of factors and the key element of the process was the gravity thickening sludge holding tank.

## Methods

### Sludge cultivation

**Figure 7:**
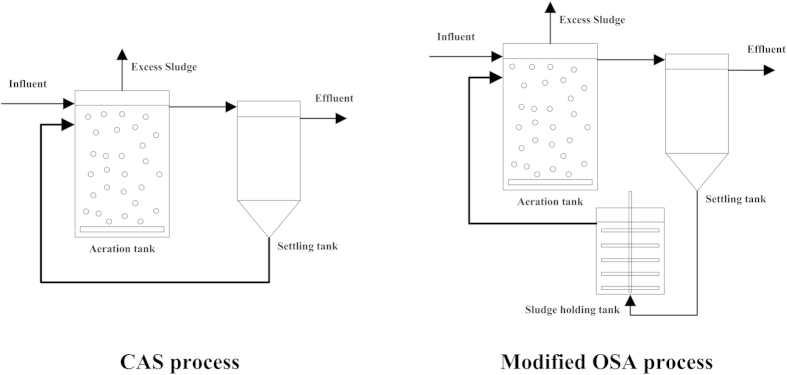
Schematic diagram of the CAS and the modified OSA processes.

Two continuous-flow systems were used to cultivate activated sludge, as shown in Fig. 7 and outlined in Table 2. Sludge taken from a local wastewater treatment plant was used as the seed in both systems to start the cultivation. The influent COD concentration increased stepwise from 50 to 250 mg/l within 2-month cultivation period.

Since PAOs enrichment had not been found in our previous study^7^, a high influent TP concentration (51 mgP/l) was employed to promote the growth of the PAOs and then improve the phosphorus removal because the P removal can be improved by increasing the influent COD/P ratio^32^. The hydraulic retention time (HRT) and dissolved oxygen (DO) level of the aeration tank were kept at 6.9 h and around 7 mg/l in each system. The TSS in the aeration tank of each system was maintained at 1700 ∼ 1800 mg/l. Temperature and pH were controlled at 20 °C and around 7.4. COD, NH_4_^+^-N, NO_3_^−^-N, TN, TP, TSS and VSS were analyzed according to *Standard Methods*^33^.

Synthetic wastewater with the following composition was used to feed to each system: Starch 203.11 mg/l, peptone 83.07 mg/l, beef extract 38.39 mg/l, NH_4_Cl 60.00 mg/l, KH_2_PO_4_ 118.00 mg/l, Na_2_HPO_4_ 123.00 mg/l, CaCl_2_·2H_2_O 11.52 mg/l, MgCl_2_·6H_2_O 150.00 mg/l, NaHCO_3_ 125.00 mg/l, and 1.0 ml/l nutrient solution. The nutrient solution consisted of the following compounds per liter: 3000 mg of EDTA-DS, 45 mg of H_3_BO_3_, 450 mg of FeCl_3_·6H_2_O, 9 mg of CuSO_4_·5H_2_O, 50 mg of (NH_4_)_6_Mo_7_O_24_·4H_2_O, 54 mg of KI, 36 mg of MnCl_2_·4H_2_O, 36 mg of ZnSO_4_·7H_2_O, 45 mg of CoCl_2_·6H_2_O, 50 mg of Al_2_(SO_4_)_3_·18H_2_O and 1.4 mg of NiSO_4_·6H_2_O.

### Operation of a modified OSA system

After 2-month cultivation, the effluent concentrations and the excess sludge production became stable. In the modified OSA system, neither pure nitrogen gas was injected nor reductant was dosed into the sludge holding tank to control the ORP level. Re-circulated sludge was pumped from the bottom of the settling tank to the bottom of the sludge holding tank and then overflowed back to the aeration tank. Sludge in the sludge holding tank was thickened by the gravity and mixed with a low speed paddle agitator with 1.5 rpm to distribute sludge equably. The sludge concentration decreased gradually from the bottom to the top in the sludge holding tank and the average sludge concentration was about 43069 mg/l. The HRT in the sludge holding tank was controlled at 10.6 h. The substrate loading, DO, Temperature and TSS concentration in the modified OSA system were maintained at the same levels as in the CAS system.

### Batch experiments

Since the sludge concentration in the sludge holding tank was the key element of the modified OSA process, the sludge withdrawn from the aeration tank of the modified OSA system was used to perform batch experiments to confirm the effect of different initial sludge concentration on sludge reduction. The sludge was transferred to one batch reactor after washed several times to clean the remained soluble substrate and then remove background interference because the sludge itself was the main concern. The modified OSA sludge was settled through centrifugation and the supernatant was withdrawn to reach a high TSS level. After that, the modified OSA thickened sludge was transferred to a batch reactor and left under anaerobic condition without external substrate supply for 22 hours with 3 different initial TSS levels. A longer reaction time (22 h) was employed to enhance the sludge reduction effect. The initial and final COD, NH_4_^+^-N, TP and TSS were monitored. All these experiments were conducted under 20 °C. All measurements in the batch experiments were repeated three times.

## Additional Information

**How to cite this article**: Wang, J. *et al.* A modified oxic-settling-anaerobic activated sludge process using gravity thickening for excess sludge reduction. *Sci. Rep.* **5**, 13972; doi: 10.1038/srep13972 (2015).

## Supplementary Material

Supplementary Information

## Figures and Tables

**Table 1:** Comparison of the excess sludge reduction with different OSA processes.

Parameter	Unit	Chudoba^a^	Saby^b^	Wang^c^	This study
Organic loading	gCOD/gTSS·d^−1^	0.33 ∼ 0.92	0.66	0.41	0.48
Average TSS in sludge holding tank	gTSS/l	2 ∼ 5	9	8 ∼ 12	43
Average ORP level in sludge holding tank	mV	−250	+100 ∼ −250	−250	−170
SRT	d	5 ∼ 12	19 ∼ 30	10	115
Y_obs_	gTSS/gCOD	0.24 ∼ 0.25	0.32 ∼ 0.18	0.24	0.24
Excess sludge reduction (calculated as TSS)	%	0 ∼ 36.8	23.4 ∼ 51.1	44.3	33.3

^a^Calculated from Chudoba *et al.*^5^.

^b^Calculated from Saby *et al.*^7^.

^c^Calculated from Wang *et al.*^9^.

**Table 2:** Operation parameters of the CAS process the modified OSA process.

Parameter	Unit	CAS	Modified OSA
Influent flow rate	L/d	17	17
Waste flow rate	L/d	0.7	0.5
Recirculation flow rate	L/d	23.3	5.2
Volume of aeration tank	L	4.86	4.86
Volume of settling tank	L	2.27	2.27
Volume of sludge holding tank	L	—	2.3
Sludge retention time	d	6.97	115.04
Hydraulic retention time in aeration tank	h	6.86	6.86
Hydraulic retention time in settling tank	h	1.38	2.51
Hydraulic retention time in holding tank	h	—	10.62
Average sludge concentration in aeration tank	mg/l	1769	1737
Average sludge concentration in sludge holding tank	mg/l	—	43069
Average pH in aeration tank	—	7.4	7.5
Average ORP level in sludge holding tank	mV	—	−170
Average influent COD	mgCOD/l		248
Average influent NO_3_^−^-N	mgN/L		4
Average influent NH_4_^+^-N	mgN/L		18
Average influent TN	mgN/L		32
Average influent TP	mgP/L		51
