# Supplementary material for: A modified oxic-settling-anaerobic activated sludge process using gravity thickening for excess sludge reduction
Source: Sci Rep. 2015 Sep 9;5:13972. doi: 10.1038/srep13972 (PMC4563565; doi:10.1038/srep13972)
Supplement: Supplementary Information [file srep13972-s1.pdf]

A modified oxic-settling-anaerobic activated sludge process using gravity thickening for excess sludge reduction

Jun Wang, Shi-Yu Li, Feng Jiang, Ke Wu, Guang-Li Liu, Hui Lu, and Guang-Hao Chen

Supplementary Information

To better understand the sludge reduction mechanism, the sludge withdrawn from the aeration tank of the CAS and the modified OSA systems were used to perform 2 batch experiments to evaluate the substrate source in the sludge holding tank and the sludge decay behavior.

For each batch experiment, the sludge was transferred to one batch reactor after washed several times to remove the remained soluble substrate and then remove background interference. 1) The CAS and the modified OSA sludge were transferred to two batch reactors after washed and left under anaerobic condition without external substrate supply for 12 hours. COD, NH_4^+-N, TP and TSS were monitored over time. 2) The CAS and the modified OSA sludge were aerated for 7 days after washed to ensure the remained particulate biodegradable organics (X_S) depleted and then transferred to two batch reactors after washed several times again, the sludge were left under anaerobic condition without external substrate supply for 12 hours. COD, NH_4^+-N, TP and TSS were monitored over time. All these experiments were conducted under 20℃.

During anaerobic treatment in the 1st batch experiment, COD, TP and NH_4^+-N concentrations increased gradually with TSS concentration decreased, as shown in Fig. 1. In this study, the average ratio between the particle COD and soluble COD in the influent of both systems was 1.29, more than half of the influent substrates existed as particulate form. Among these, X_S will be converted to soluble form by hydrolysis before utilized by bacteria. Since hydrolysis is a relatively slow process, X_S cannot be completely degraded in the aeration tank, hydrolysis will continue in the sludge holding tank which made the remained X_S as a hidden substrate source for those anoxic/anaerobic bio-processes and the amount of excess sludge will be reduced accordingly. However, similar behaviors were found in the 2nd batch experiment (as shown in Fig. 2) meant that hydrolysis of the remained X_S might not be the only substrate source. The 2nd batch experiment was conducted under the same conditions as in the 1st batch experiment except sludge were pre-aerated 7 days to make sure the remained Xs had been depleted. The endogenous oxygen uptake rates (OUR) dropped below 1 mgO_2/l/h after aerated. It was found that the COD and TP kept releasing after aerated pretreatment while the NH_4^+-N concentration was close to zero. These indicated that the biomass itself can also be used as substrate under such condition. Therefore, the biomass decay process under anaerobic condition cannot be

neglected, which was not included in some activated sludge model[1]. These results will help further dynamic modeling developing and better understanding the sludge decay behavior.

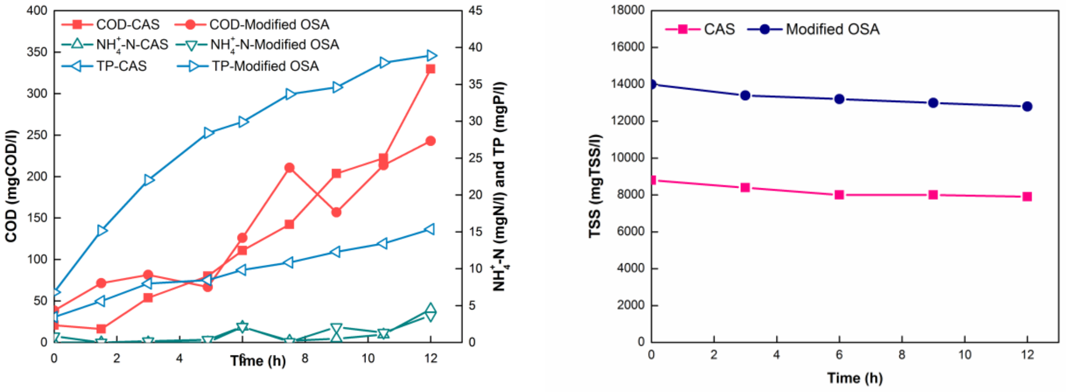

Figure 1. Concentration of COD, NH_4^+-N, TP and TSS during the 1st batch experiment

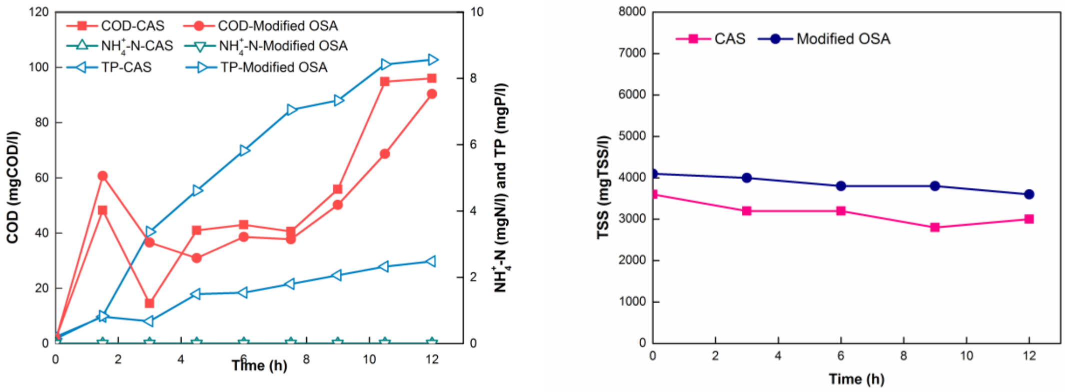

Figure 2. Concentration of COD, NH_4^+-N, TP and TSS during the 2nd batch experiment

References

1 Rieger, L., Koch G. & Kuhni M. The EAWAG Bio-P module for activated sludge model NO.3. *Water Res.* **35,** 3887-903 (2001).
